# Integrating Bulk and Single-Cell RNA Sequencing Identifies and Validates Lactylation-Related Signatures in Acute Kidney Injury

**DOI:** 10.3390/biomedicines14081810

**Published:** 2026-08-12

**Authors:** Heping Niu, Zhendong Tian, Ziyi Li, Shujun Shi, Rongrong Deng, Jiangwei Man, Xiaochun Zhou, Li Huang, Jianqin Wang

**Affiliations:** 1 Department of Nephrology, The Second Hospital of Lanzhou University, Lanzhou 730030, China; 2 Gansu Province Clinical Research Center for Renal Diseases, Lanzhou 730030, China; 3 Department of Urology, The Second Hospital of Lanzhou University, Lanzhou 730030, China

**Keywords:** lactylation, acute kidney injury, lactate metabolism, single-cell RNA sequencing, biomarkers

## Abstract

**Background**: Acute kidney injury (AKI) is a severe clinical syndrome characterized by metabolic stress and profound inflammation. However, the landscape of lactylation-associated molecular alterations and their potential relevance in AKI remain incompletely understood. **Methods**: Bulk transcriptomes (GSE30718) were analyzed using the limma package, weighted gene co-expression network analysis (WGCNA), and consensus clustering to characterize AKI-associated molecular patterns linked to lactylation-associated signatures. Hub genes, prioritized through least absolute shrinkage and selection operator (LASSO) regression and the random forest algorithm, were integrated into a diagnostic nomogram and evaluated in an external cohort (GSE139061). Immune infiltration analysis was performed, and single-cell RNA sequencing data (GSE183276) were used to resolve cell-type-specific expression patterns of the hub genes. A cisplatin-induced murine AKI model validated global protein lactylation and hub gene expression by immunohistochemistry and Western blot. **Results**: Three hub genes (CKLF, ACLY, and SLC13A3) reliably discriminated AKI from controls (training AUC = 0.897). Their diagnostic performance varied in the external validation cohort. These genes correlated significantly with diverse immune infiltrates. Single-cell analysis localized CKLF predominantly to immune cells, ACLY broadly across renal populations, and SLC13A3 to proximal tubules. In vivo validation demonstrated increased global protein lysine lactylation levels in injured kidneys and confirmed expression alterations of CKLF, ACLY, and SLC13A3 consistent with transcriptomic observations. **Conclusions**: Integrating transcriptomic analysis with in vivo experimental validation, this study identified CKLF, ACLY, and SLC13A3 as candidate lactylation-associated signatures linked to immune and metabolic alterations in AKI.

## 1. Introduction

Acute kidney injury (AKI) is a common and serious clinical syndrome defined by a rapid decline in renal function over hours to days, with retention of nitrogenous waste products, disturbance of fluid and electrolyte balance, and a fall in urine output [1]. It arises frequently in hospitalized patients, and particularly in those who are critically ill, and is associated with prolonged hospitalization, higher health care costs, and substantial mortality [2]. Beyond the acute episode, survivors of AKI carry an elevated risk of incomplete renal recovery and of progression to chronic kidney disease [3,4]. Although supportive care has improved, no specific pharmacological therapy reliably reverses established injury, and management remains largely confined to the avoidance of further insult and to renal replacement therapy. This gap highlights the need to define the molecular mechanisms that drive AKI and to identify candidate biomarkers and targets that could enable earlier recognition and intervention.

The pathophysiology of AKI is multifactorial and involves tubular epithelial cell injury, microvascular dysfunction, sterile inflammation, and metabolic disturbance [5,6]. Among these processes, metabolic reprogramming has emerged as an important but incompletely understood contributor to renal injury. Under ischemic and hypoxic conditions, renal tubular cells, which normally rely heavily on oxidative phosphorylation, shift toward anaerobic glycolysis [7,8]. This metabolic switch helps to sustain ATP production but also leads to the accumulation of lactate within injured tissue and in the systemic circulation. Once viewed simply as a glycolytic end product, lactate is now recognized as a versatile signaling molecule that shapes immune function, gene transcription, and cellular metabolism [9,10,11,12,13].

A key mechanism linking lactate to gene regulation is protein lactylation, a post-translational modification in which lactate-derived lactyl groups are covalently added to lysine residues on histone and non-histone proteins [14,15]. Histone lactylation reshapes chromatin accessibility and promotes the transcription of inflammatory and reparative gene programs, whereas non-histone lactylation modulates the activity, stability, and interactions of metabolic enzymes and signaling proteins. Growing evidence indicates that lactylation operates as a metabolic and epigenetic switch during ischemia–reperfusion injury in several organs, including the heart, brain, and liver [16,17,18]. In the kidney, lactate metabolism and lactylation have been implicated in tubular repair and inflammatory signaling after ischemic injury [19,20]. Despite these advances, the global landscape of lactylation-associated signature regulation in AKI has not been systematically characterized.

In the present study, we integrated bulk and single-cell transcriptomic data with a curated catalogue of lactylation-associated signatures to delineate the lactylation-associated molecular features of AKI. By combining differential expression analysis, weighted gene co-expression network analysis, consensus clustering, and machine learning, we identified and prioritized lactylation-associated signatures associated with AKI, evaluated their diagnostic value, examined their relationship with immune cell infiltration, and resolved their cell-type-specific expression at single-cell resolution. We further validated the bioinformatic findings in a cisplatin-induced mouse model of AKI. This framework connects lactate metabolism to the transcriptional and immune landscape of AKI and nominates candidate regulators for further mechanistic and translational study.

## 2. Materials and Methods

### 2.1. Data Acquisition and Preprocessing

Transcriptomic datasets were retrieved from the Gene Expression Omnibus (GEO) database (https://www.ncbi.nlm.nih.gov/geo/, accessed on 20 October 2025). The GSE30718 dataset, which served as the training cohort, comprised 47 human kidney samples, including 28 specimens with AKI and 19 control specimens [21]. An independent bulk dataset, GSE139061, comprising 39 AKI samples and 9 control samples, was utilized as the external validation cohort [22]. Furthermore, to map the cellular distribution of the candidate genes, we utilized the single-cell RNA sequencing dataset GSE183276, which included 12 AKI and 20 control kidney samples [23].

A catalogue of lactylation-associated signatures was assembled from the Molecular Signatures Database (MSigDB; https://www.gsea-msigdb.org/gsea/index.jsp, accessed on 23 October 2025) and from peer-reviewed studies of protein lactylation. After removal of duplicate entries, 734 unique lactylation-associated signatures were retained for downstream analysis.

### 2.2. Differential Expression and Functional Enrichment Analysis

Differentially expressed genes (DEGs) between AKI and control samples in the training cohort were identified with the limma package. Multiple testing correction was performed using the Benjamini–Hochberg false discovery rate (FDR) method, and genes with an adjusted *p* value < 0.05 and |log2 fold change| ≥ 0.585 were considered statistically significant. The overall distribution of these genes was then illustrated using volcano plots and heatmaps. Functional annotation of the DEGs was performed with the clusterProfiler package [24], including Gene Ontology (GO) analysis across the biological process, cellular component, and molecular function categories, and Kyoto Encyclopedia of Genes and Genomes (KEGG) pathway analysis. Gene set enrichment analysis (GSEA) was additionally carried out by ranking all genes according to their differential expression and assessing the enrichment of predefined gene sets.

### 2.3. Weighted Gene Co-Expression Network Analysis

To identify co-expressed gene modules associated with AKI, weighted gene co-expression network analysis (WGCNA) was performed with the WGCNA package. Genes with a coefficient of variation greater than 0.05 were retained for network construction. A soft-thresholding power of 5 was selected to satisfy the scale-free topology criterion (R2 > 0.9). Co-expression modules were defined by hierarchical clustering followed by dynamic tree cutting, and module eigengenes were correlated with the AKI and control traits. The module showing the strongest correlation with AKI was designated as the hub module, and its member genes with high module membership (MM > 0.7) and gene significance (GS > 0.2) were extracted for subsequent analysis.

### 2.4. Consensus Clustering

Consensus clustering of the AKI samples was performed on the basis of the identified Lactylation-associated signatures using the ConsensusClusterPlus package. Clustering was carried out with the partitioning around medoids algorithm over 1000 resampling iterations. The optimal number of clusters was determined by inspecting the consensus matrix, the cumulative distribution function, and the delta area plot, and the resulting subtypes were visualized by principal component analysis (PCA).

### 2.5. Machine Learning

Candidate-gene prioritization and random-forest performance evaluation were conducted as separate steps. First, four prespecified lactylation-associated candidate genes (RALYL, CKLF, ACLY, and SLC13A3) identified from the integrative transcriptomic analysis were evaluated in the GSE30718 training cohort using least absolute shrinkage and selection operator (LASSO) logistic regression (glmnet package) and random forest classification (randomForest package). The optimal penalty parameter (λ) for LASSO regression was selected based on the cross-validation error curve, and genes with nonzero coefficients were retained. Random-forest variable importance was assessed using the mean decrease in the Gini index. Genes consistently selected by both algorithms were retained as final candidate signatures for subsequent analyses.

To obtain an optimism-reduced estimate of random-forest performance, an independent internal validation analysis was performed using all 47 GSE30718 samples (28 AKI and 19 controls) with stratified five-fold cross-validation repeated 10 times. This internal validation analysis was performed solely to estimate model generalizability and was independent of the predefined candidate signature selection procedure. For each repeat, AKI and control samples were randomized separately and assigned to five folds. Importantly, feature selection was performed exclusively within the training folds to prevent information leakage. All genes in the expression matrix were ranked according to the standardized mean difference between AKI and control groups, and the top 10 genes were used for model training within each fold. The held-out fold was used only for model evaluation.

Each random forest consisted of 1000 trees with predefined hyperparameters, including an mtry value of 3 and a minimum terminal-node size of 8. Class-balanced bootstrap sampling was applied, and a fixed random seed (20260731) was used to ensure reproducibility. Out-of-fold predicted probabilities were generated for each sample, and the ROC curve, AUC, 95% confidence interval, accuracy, sensitivity, specificity, and Brier score were calculated using the pROC package. These procedures were implemented to minimize information leakage and reduce the risk of overfitting.

### 2.6. External Validation and Diagnostic Evaluation

The differential expression of the hub genes was examined in both the training and the external validation cohorts. The diagnostic performance of each gene was assessed by receiver operating characteristic (ROC) analysis using the pROC package, with the area under the curve (AUC) used as the measure of discrimination.

### 2.7. Nomogram Construction

A diagnostic nomogram incorporating the hub genes was constructed with the rms package. The predictive performance of the nomogram and of the individual genes was evaluated by ROC analysis. Calibration curves, decision curve analysis (DCA), and a clinical impact curve were generated to assess calibration, net clinical benefit, and clinical applicability.

### 2.8. Immune Cell Infiltration Analysis

The relative abundance of 22 immune cell types in each sample was estimated with the CIBERSORT algorithm. Differences in immune cell composition between AKI and control samples were assessed with the Wilcoxon test. Correlations among immune cell types and between the hub genes and immune cell types were computed and visualized.

### 2.9. Single-Cell RNA Sequencing Analysis

The GSE183276 dataset was processed with the Seurat package [25]. Low-quality cells were excluded on the basis of standard quality-control thresholds for the number of detected genes and the mitochondrial transcript content. Gene expression was normalized, and the 2000 most variable genes were used for scaling and principal component analysis. Batch effects between samples were corrected with Harmony [26], after which the cells were embedded and visualized by uniform manifold approximation and projection (UMAP) and grouped into clusters. Marker genes identified with the FindAllMarkers function were used to annotate the clusters into known renal cell types, and the expression of the hub genes was examined across cell types and between the control and AKI groups.

### 2.10. Ethical Compliance

All animal experiments were reviewed and authorized by the Animal Ethics Committee of Lanzhou University Second Hospital. Experimental animals were housed, handled, and treated in accordance with the national standards for laboratory animal welfare and ethical practice established by the Ministry of Science and Technology of the People’s Republic of China. The design, conduct, and reporting of the in vivo experiments also followed the ARRIVE recommendations to enhance methodological transparency and reproducibility.

### 2.11. Animal Experiments

Male C57BL/6 mice aged 6–8 weeks and weighing 20–25 g were obtained from the Lanzhou Veterinary Research Institute. After one week of acclimatization, the mice were randomly assigned to two groups: the control group and the cisplatin-induced acute kidney injury (AKI) group, with six mice in each group (*n* = 6 per group). To establish the AKI model, mice in the cisplatin group received a single intraperitoneal injection of cisplatin (Cat. No. S1166; Selleck, Shanghai, China) at a dose of 20 mg/kg. Mice in the control group were administered an equal volume of sterile normal saline via intraperitoneal injection. Seventy-two hours after cisplatin or saline administration, the mice were deeply anesthetized with sodium pentobarbital and subsequently euthanized by cervical dislocation. Death was confirmed by the absence of spontaneous respiration and heartbeat. Blood samples and kidney tissues were then collected for subsequent analyses. All mice were maintained under standard laboratory conditions, including a controlled temperature of 22–24 °C, relative humidity of 50–60%, and a 12 h light/dark cycle. Standard chow and water were provided ad libitum throughout the experimental period. All animal experiments were approved by the Animal Ethics Committee of the Second Hospital of Lanzhou University and reported in accordance with the ARRIVE guidelines [27].

### 2.12. Measurement of Serum Creatinine and Blood Urea Nitrogen

Mouse blood samples were collected from the retro-orbital venous plexus and allowed to stand at room temperature for 30 min to clot (*n* = 6 per group). The samples were then centrifuged at 3000× *g* for 10 min at 4 °C to separate the serum. Serum creatinine (Scr) levels were measured using the alkaline picrate method based on the Jaffe reaction. Briefly, creatinine reacts with alkaline picrate to form a colored complex, and the color intensity is proportional to the creatinine concentration in the serum. Blood urea nitrogen (BUN) levels were determined using an enzymatic colorimetric method based on the urease–glutamate dehydrogenase coupled reaction. In this assay, urea is hydrolyzed by urease to generate ammonium ions, which participate in the glutamate dehydrogenase-catalyzed reaction, and the resulting absorbance change reflects the BUN concentration. The absorbance values were read using an automatic biochemical analyzer (Rayto Life Science Co., Ltd., Shenzhen, China), and the levels of Scr and BUN were used to evaluate renal function in mice.

### 2.13. Histopathological Staining

Kidney tissues were fixed in 4% paraformaldehyde, dehydrated through a graded ethanol series, embedded in paraffin, and sectioned at a thickness of 4 μm (*n* = 6 per group). For histopathological evaluation, kidney sections were subjected to hematoxylin and eosin (H&E) staining and periodic acid–Schiff (PAS) staining using commercial kits (Solarbio, Beijing, China) according to the manufacturer’s instructions. H&E staining was used to assess general renal morphology, including tubular epithelial injury, tubular dilation, inflammatory cell infiltration, and structural disruption. PAS staining was performed to evaluate tubular brush border integrity, basement membrane alterations, and glycogen/polysaccharide-related pathological changes. After staining, sections were dehydrated, cleared, and mounted with neutral resin. Representative images were acquired using a light microscope for subsequent histological analysis.

### 2.14. Immunohistochemistry (IHC)

Immunohistochemistry (IHC) was performed to detect pan-lysine lactylation (Pan-Kla) expression in kidney tissues (*n* = 6 per group). Paraffin-embedded kidney sections were deparaffinized in xylene, rehydrated through a graded ethanol series, and subjected to antigen retrieval. Endogenous peroxidase activity was blocked with 3% hydrogen peroxide, followed by blocking with normal serum to reduce nonspecific binding. The sections were then incubated overnight at 4 °C with a primary antibody against Pan-Kla (1:200; PTM-1401RM, PTMBio, Hangzhou, China). After washing, the sections were incubated with an appropriate horseradish peroxidase-conjugated secondary antibody according to the manufacturer’s instructions. Immunoreactive signals were visualized using diaminobenzidine (DAB), and the nuclei were counterstained with hematoxylin. Finally, the sections were dehydrated, cleared, mounted with neutral resin, and imaged under a light microscope.

### 2.15. Western Blot

Total proteins were extracted from kidney tissues using RIPA lysis buffer supplemented with protease inhibitor (*n* = 6 per group). The lysates were centrifuged at 12,000× *g* for 15 min at 4 °C, and the supernatants were collected. Protein concentrations were determined using a BCA protein assay kit according to the manufacturer’s instructions. Equal amounts of protein from each sample were separated by SDS-PAGE and subsequently transferred onto PVDF membranes. After blocking with 5% bovine serum albumin for 1 h at room temperature, the membranes were incubated overnight at 4 °C with the following primary antibodies: Pan-Kla (PTM-1401RM, PTMBio, Hangzhou, China), ACLY (15421-1-AP, Proteintech, Wuhan, China), SLC13A3 (26184-1-AP, Proteintech, Wuhan, China), and CKLF (21017-1-AP, Proteintech, Wuhan, China). After washing with TBST, the membranes were incubated with the corresponding secondary antibody (S0001, Affinity, Changzhou, China) for 1 h at room temperature. Protein bands were visualized using an enhanced chemiluminescence detection system. The band intensities were quantified using ImageJ software (version 1.53) and normalized to the corresponding loading control.

### 2.16. Statistical Analysis

All quantitative data were analyzed with R software (v4.2.2) and GraphPad Prism 8.2.1. Results are shown as the mean ± SD. For comparisons between two groups, an unpaired *t*-test was applied. Differences were considered statistically significant at *p* < 0.05, with significance levels indicated as * *p* < 0.05.

## 3. Results

### 3.1. Identification of DEGs and Functional Characterization

**Figure 1:**
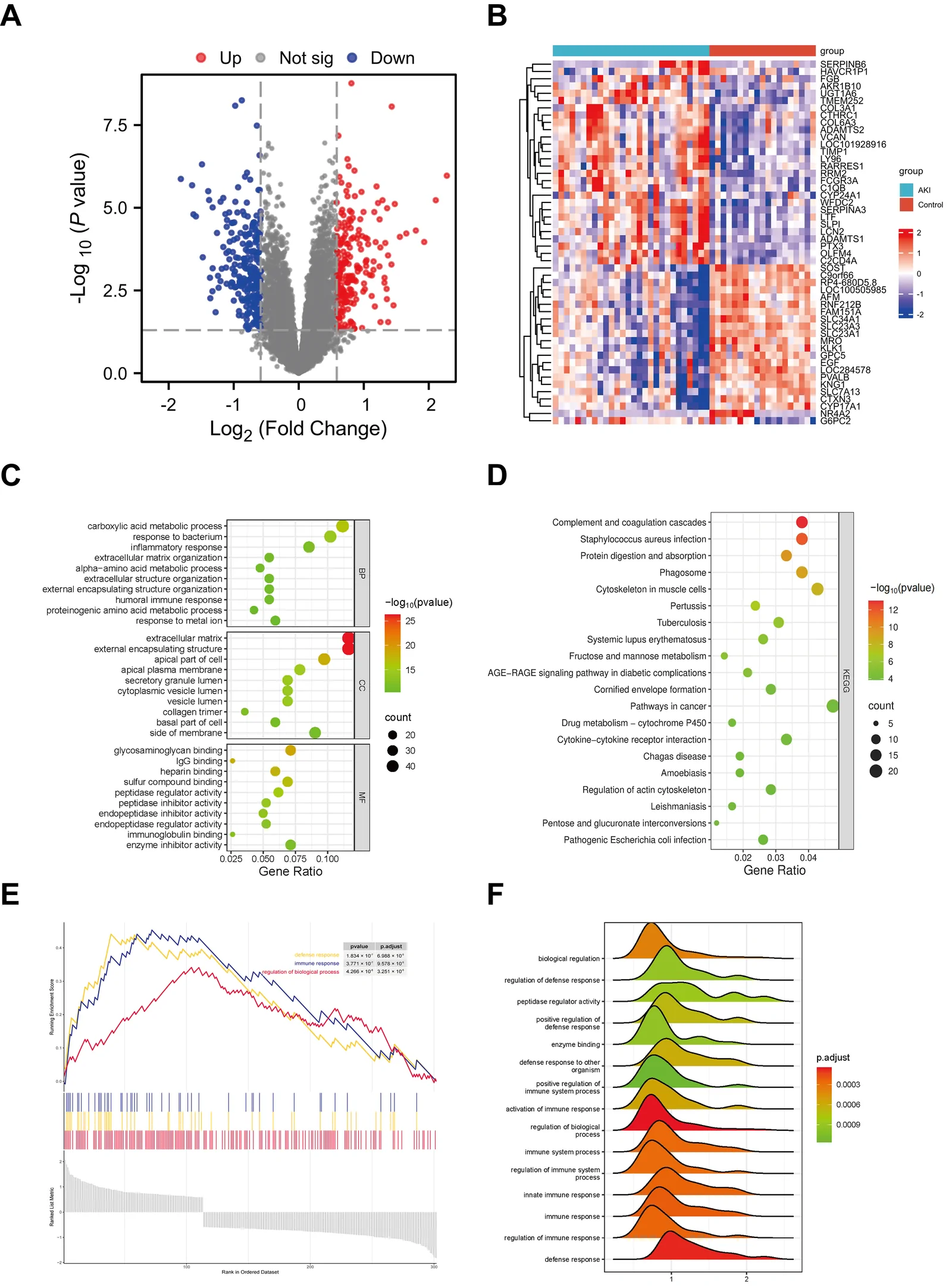
Differential expression and functional enrichment analysis in the training cohort (GSE30718). (**A**) Volcano plot of genes differentially expressed between AKI and control kidney samples (red, upregulated; blue, downregulated; gray, not significant). (**B**) Heatmap of representative DEGs across the AKI and control groups. (**C**) GO enrichment of DEGs across the biological process (BP), cellular component (CC), and molecular function (MF) categories. (**D**) KEGG pathway enrichment of DEGs. (**E**) GSEA of the most significantly enriched gene sets. (**F**) Ridge plot summarizing the GSEA enrichment distributions.

Comparison of AKI and control kidney samples in the training cohort identified 441 DEGs, with 201 upregulated and 240 downregulated (Figure 1A). The expression patterns of representative DEGs clearly separated the AKI and control groups (Figure 1B). GO analysis showed that the DEGs were enriched in biological processes related to the inflammatory and humoral immune response, the response to bacterium, extracellular matrix organization, and carboxylic acid and amino acid metabolism (Figure 1C). In the cellular component and molecular function categories, terms linked to the extracellular matrix, secretory granules, and peptidase regulator and inhibitor activity were prominent. KEGG analysis highlighted pathways including complement and coagulation cascades, protein digestion and absorption, phagosome, cytokine–cytokine receptor interaction, and several infection- and metabolism-related pathways (Figure 1D). GSEA reinforced these observations and showed significant enrichment of immune and defense response programs (Figure 1E,F). Taken together, these results indicate that AKI is accompanied by coordinated activation of inflammatory signaling, extracellular matrix remodeling, and metabolic reprogramming.

### 3.2. Identification of an AKI-Associated Co-Expression Module

**Figure 2:**
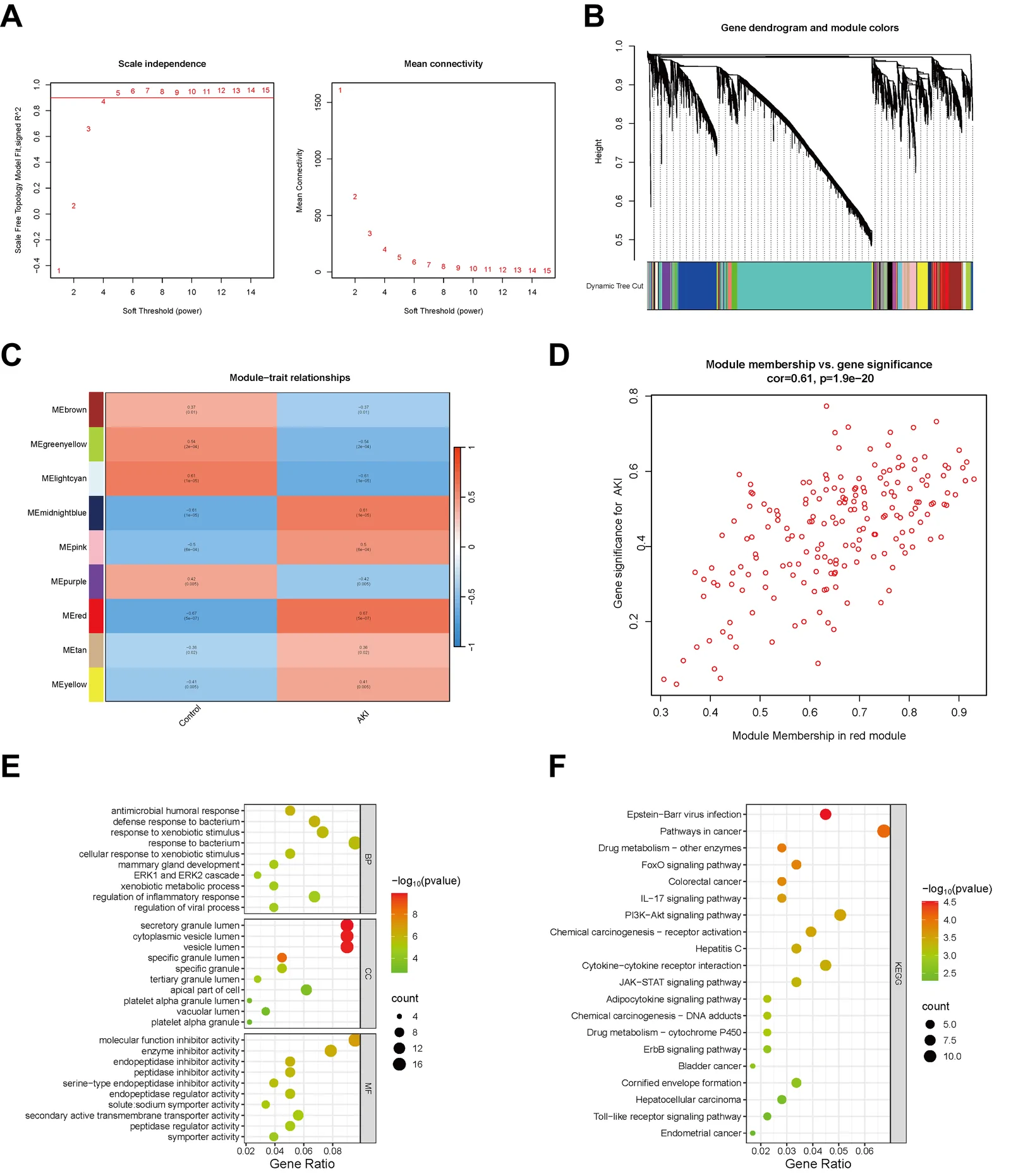
Weighted gene co-expression network analysis. (**A**) Scale-free topology fit index and mean connectivity across candidate soft-thresholding powers, with the optimal power selected for network construction. (**B**) Gene dendrogram showing hierarchical clustering of genes and module color assignments. (**C**) Module–trait relationship heatmap; the red module showed the strongest positive correlation with AKI. (**D**) Scatter plot showing the relationship between module membership and gene significance in the AKI-associated red module. (**E**) GO enrichment of the red module genes. (**F**) KEGG pathway enrichment of the red module genes.

To uncover gene modules associated with AKI, we built a weighted co-expression network using genes with high expression variability. A soft-thresholding power of 5 yielded a scale-free network (Figure 2A), and dynamic tree cutting partitioned the genes into distinct modules (Figure 2B). Correlation of the module eigengenes with the sample traits showed that the red module had the strongest positive association with AKI (r = 0.67, *p* = 5 × 10^−7^; Figure 2C). Within this module, module membership was strongly correlated with gene significance for AKI (cor = 0.61, *p* = 1.9 × 10^−20^; Figure 2D), and 187 hub genes were retained. Functional analysis of the red module genes revealed enrichment in defense and antimicrobial humoral responses, the regulation of inflammatory responses, and transmembrane transporter activity, including solute: sodium symporter activity (Figure 2E). KEGG analysis identified inflammatory and signaling pathways such as IL-17 signaling, cytokine–cytokine receptor interaction, JAK-STAT signaling, PI3K-Akt signaling, FoxO signaling, and Toll-like receptor signaling (Figure 2F). These findings are consistent with the differential expression analysis and point to inflammation and altered solute transport as features of the AKI-associated module.

### 3.3. Identification of Lactylation-Associated Signatures and Consensus Molecular Subtypes

**Figure 3:**
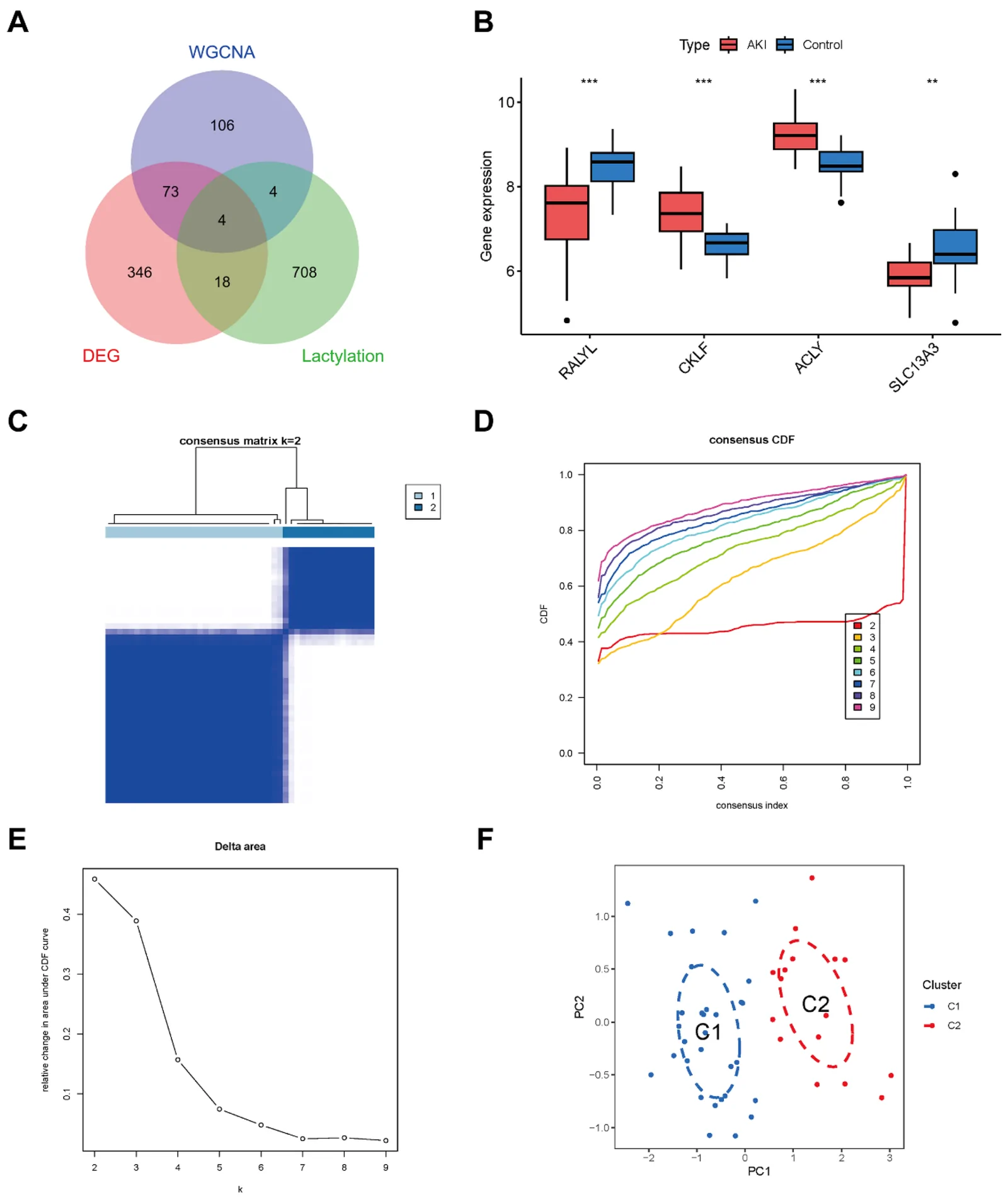
Identification of lactylation-associated signatures and consensus molecular subtypes. (**A**) Venn diagram of the overlap among DEGs, red module genes, and lactylation-associated signatures. (**B**) Expression of the four candidate lactylation-associated signatures in AKI versus control samples. (**C**) Consensus matrix showing the optimal clustering solution at k = 2, identifying two molecular subtypes (C1 and C2). (**D**) Consensus cumulative distribution function (CDF) curves evaluating cluster stability across k = 2–9. (**E**) Delta area plot indicating the optimal number of clusters based on the relative change in the CDF area. (**F**) PCA of the two consensus clusters (C1 and C2). ** *p* < 0.01, *** *p* < 0.001.

To define lactylation-associated signatures relevant to AKI, we intersected the DEGs, the red module genes, and the curated LRG set, which yielded four shared genes: RALYL, CKLF, ACLY, and SLC13A3 (Figure 3A). These four genes were differentially expressed between AKI and control samples; CKLF and ACLY were upregulated in AKI, whereas RALYL and SLC13A3 were downregulated (Figure 3B). Using these four lactylation-associated signatures, consensus clustering of the AKI samples identified two stable molecular subtypes, designated C1 and C2 (Figure 3C). The cumulative distribution function and delta area plots supported k = 2 as the optimal number of clusters (Figure 3D,E), and PCA confirmed that the two subtypes occupied distinct regions of the expression space (Figure 3F). These results indicate that lactylation-associated signatures capture meaningful heterogeneity among AKI samples.

### 3.4. Machine Learning Identifies Three Hub Lactylation-Associated Signatures

**Figure 4:**
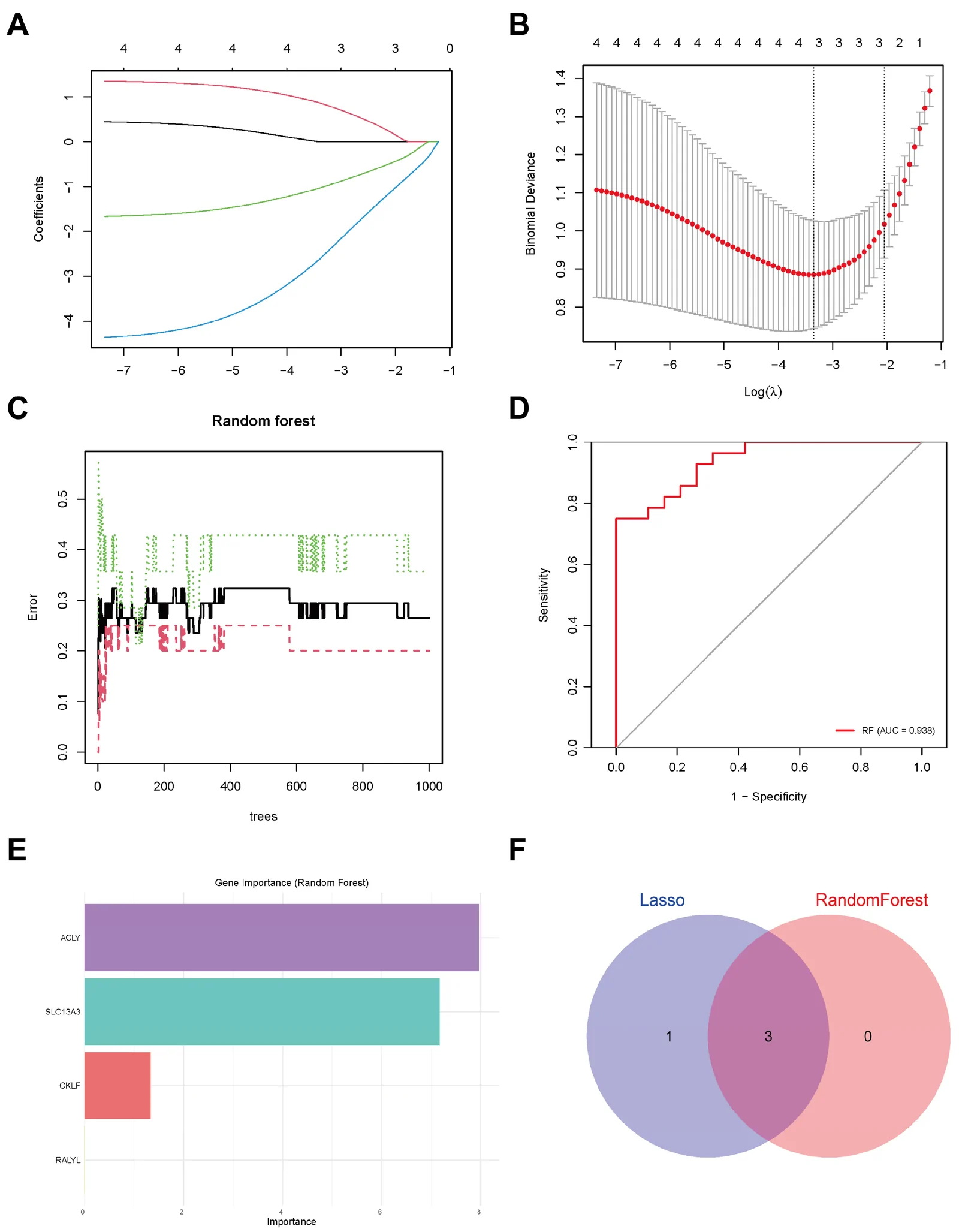
Machine-learning-based selection of hub lactylation-associated signatures. (**A**) Coefficient profiles of the LASSO regression model. (**B**) Cross-validation curve of the LASSO regression model. (**C**) Random forest error rate as a function of the number of trees. (**D**) ROC curve illustrating the classification performance of the random forest model in the training cohort. (**E**) Random forest variable importance ranking. (**F**) Venn diagram of the genes selected by LASSO and random forest.

To prioritize candidate genes associated with lactylation-associated signatures, we applied LASSO regression and random forest classification in the training cohort. LASSO regression identified a subset of candidate genes based on the optimal penalty parameter (Figure 4A,B). The random forest model showed stable performance with increasing numbers of trees and achieved an AUC of 0.938 in the training cohort (Figure 4C,D). Variable importance analysis indicated that ACLY and SLC13A3 contributed more substantially to model classification, followed by CKLF, whereas RALYL showed relatively limited contribution (Figure 4E). The intersection of genes selected by both algorithms identified CKLF, ACLY, and SLC13A3 as candidate diagnostic signatures (Figure 4F).

### 3.5. A Nomogram Based on the Hub Genes Predicts AKI

**Figure 5:**
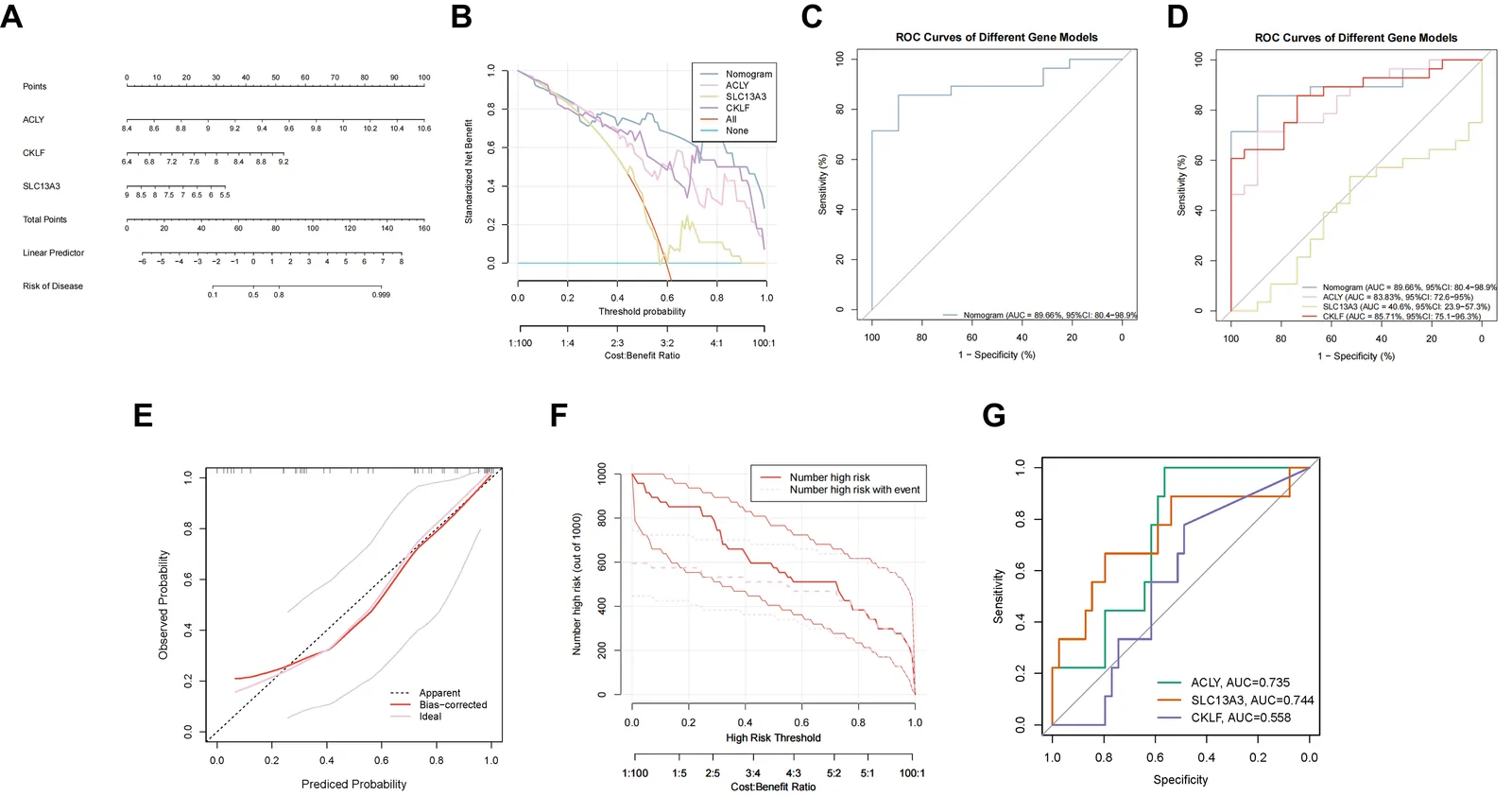
Construction and evaluation of a diagnostic nomogram. (**A**) Nomogram integrating CKLF, ACLY, and SLC13A3 to predict the risk of AKI. (**B**) Decision curve analysis of the nomogram and the individual genes. (**C**) ROC curve of the nomogram in the training cohort. (**D**) ROC curves comparing the nomogram and the individual genes in the training cohort. (**E**) Calibration curve comparing predicted and observed probabilities. (**F**) Clinical impact curve of the nomogram. (**G**) ROC curves of the individual genes in the external validation cohort.

A nomogram incorporating CKLF, ACLY, and SLC13A3 was constructed to evaluate their potential diagnostic relevance for AKI (Figure 5A). In the training cohort, the nomogram showed moderate-to-good discriminatory performance, with an AUC of 0.897 (95% CI, 0.804 to 0.989; Figure 5C,D). Among individual genes, CKLF and ACLY showed relatively higher discriminatory ability, whereas SLC13A3 exhibited limited performance in the training cohort (Figure 5D). The calibration curve indicated reasonable agreement between predicted and observed probabilities (Figure 5E). Decision curve analysis showed that the nomogram may provide additional clinical benefit compared to the individual genes across a range of threshold probabilities (Figure 5B), and the clinical impact curve supported its clinical applicability (Figure 5F). In the external validation cohort, the three genes retained variable discriminatory ability, with AUC values of 0.744 for SLC13A3, 0.735 for ACLY, and 0.558 for CKLF (Figure 5G). Notably, the diagnostic performance of CKLF decreased substantially compared with the training cohort, suggesting that its predictive capability may be influenced by cohort heterogeneity and limited sample size. Therefore, further validation using larger independent clinical cohorts is required before clinical translation.

### 3.6. Immune Cell Infiltration and Its Association with the Hub Genes

**Figure 6:**
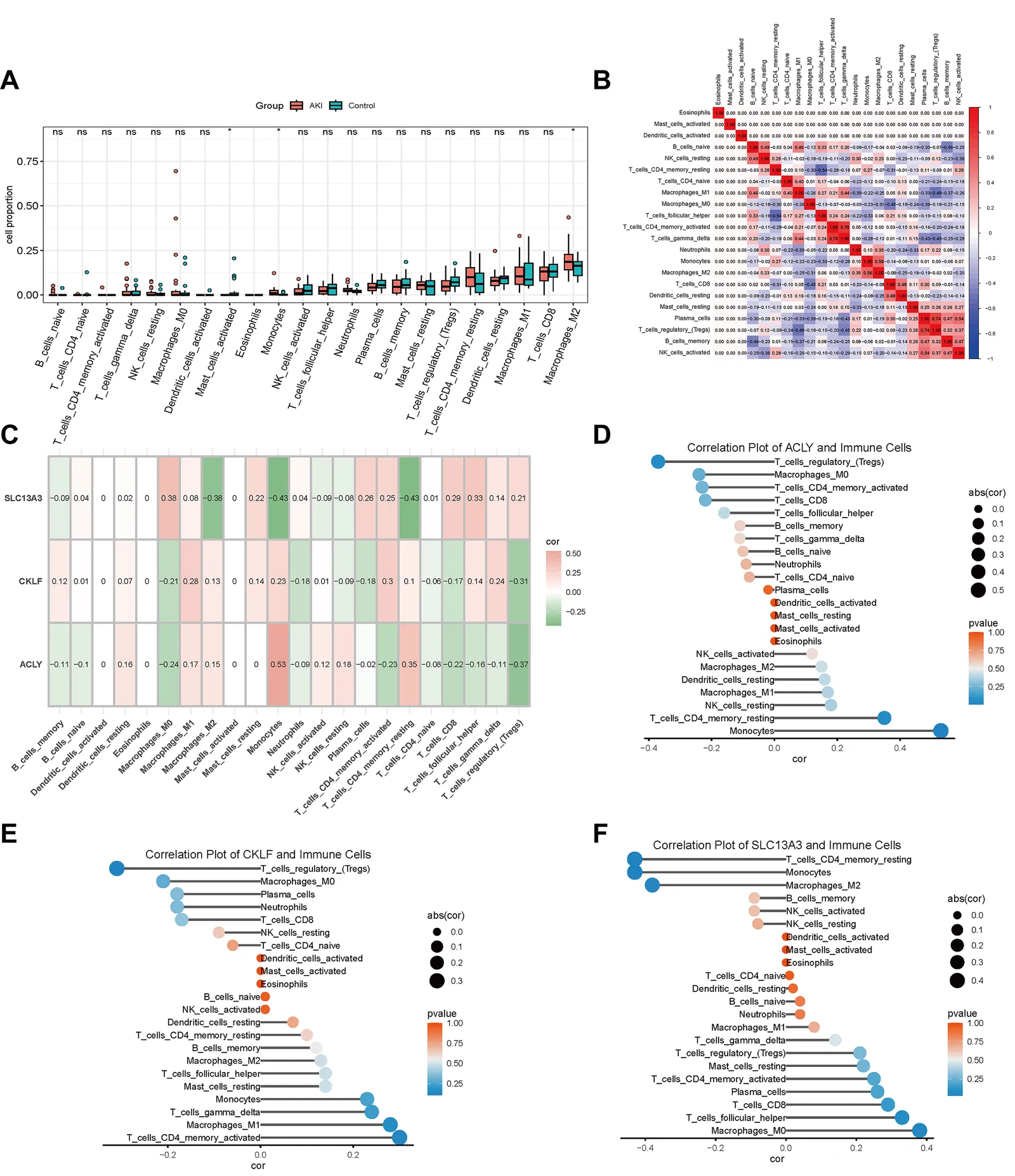
Immune cell infiltration landscape in AKI. (**A**) CIBERSORT-estimated proportions of 22 immune cell types in AKI and control samples. (**B**) Correlation matrix among the 22 immune cell types. (**C**) Heatmap of the correlations between the three hub genes and the 22 immune cell types. (**D**) Correlations of ACLY with the immune cell types. (**E**) Correlations of CKLF with the immune cell types. (**F**) Correlations of SLC13A3 with the immune cell types. ns, not significant; * *p* < 0.05.

Because inflammation is central to AKI, we characterized immune cell infiltration with CIBERSORT. The estimated composition of 22 immune cell types differed between AKI and control samples for several populations (Figure 6A), and the infiltrating cell types displayed a complex network of positive and negative correlations (Figure 6B). Correlation analysis between the hub genes and the immune cell types revealed distinct association patterns (Figure 6C). Among the strongest associations, ACLY correlated positively with monocytes and resting CD4 memory T cells and negatively with regulatory T cells (Figure 6C,D). CKLF correlated positively with activated CD4 memory T cells, M1 macrophages, gamma delta T cells, and monocytes, and negatively with regulatory T cells (Figure 6C,E). SLC13A3 correlated positively with M0 macrophages and resting CD4 memory T cells and negatively with neutrophils, activated CD4 memory T cells, and M2 macrophages (Figure 6C,F). These patterns suggest that the hub lactylation-associated signatures are linked to the activation and remodeling of the immune microenvironment in AKI.

### 3.7. Single-Cell Analysis Reveals Cell-Type-Specific Expression of the Hub Genes

**Figure 7:**
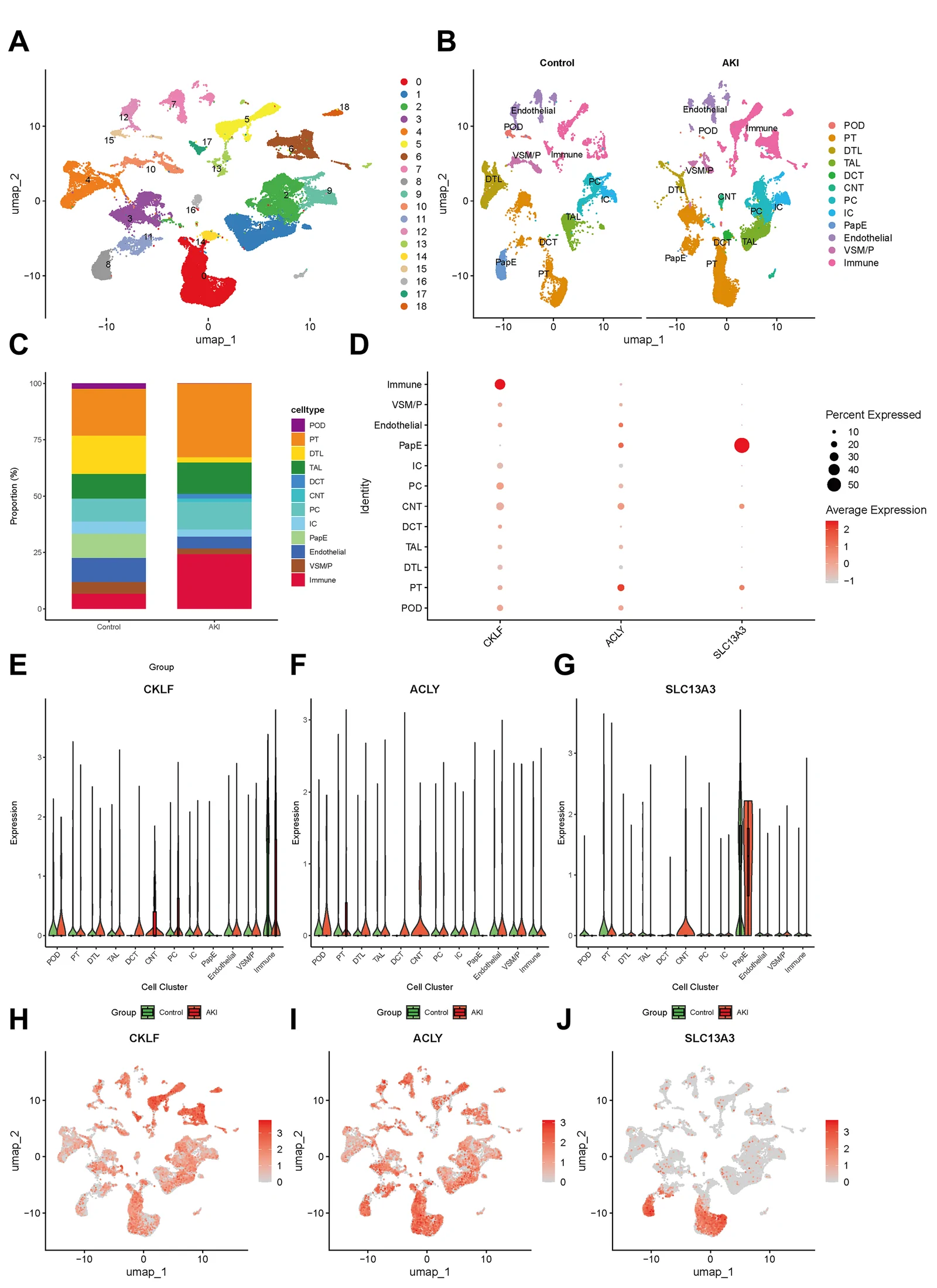
Single-cell transcriptomic analysis of AKI (GSE183276). (**A**) UMAP plot of the 19 cell clusters. (**B**) UMAP plots of the 12 annotated cell types in the control and AKI groups. (**C**) Stacked bar chart of the cell type proportions in the control and AKI groups. (**D**) Dot plot of CKLF, ACLY, and SLC13A3 expression across cell types; dot size indicates the percentage of expressing cells and color indicates the average expression. (**E**) Violin plot of CKLF expression by cell type and group. (**F**) Violin plot of ACLY expression by cell type and group. (**G**) Violin plot of SLC13A3 expression by cell type and group. (**H**) Feature plot of CKLF. (**I**) Feature plot of ACLY. (**J**) Feature plot of SLC13A3.

To resolve the cellular sources of the hub genes, we analyzed the single-cell dataset GSE183276. Unsupervised clustering grouped the cells into 19 clusters (Figure 7A), which were annotated into 12 renal cell types on the basis of canonical markers: podocytes (POD), proximal tubule cells (PT), descending thin limb (DTL), thick ascending limb (TAL), distal convoluted tubule (DCT), connecting tubule (CNT), principal cells (PCs), intercalated cells (ICs), papillary epithelium (PapE), endothelial cells, vascular smooth muscle cells and pericytes (VSM/P), and immune cells (Figure 7B). The cellular composition differed markedly between groups, with an expansion of the immune cell compartment and a contraction of the proximal tubule population in AKI relative to control (Figure 7C). Examination of the hub genes across cell types showed cell-type-restricted expression patterns (Figure 7D). CKLF was expressed most highly in immune cells and tended to increase in AKI (Figure 7E,H). ACLY was broadly expressed across cell types with a relatively uniform distribution (Figure 7F,I). SLC13A3 was largely confined to proximal tubule cells and was reduced in AKI compared with control (Figure 7G,J). These single-cell observations indicate that the three hub lactylation-associated signatures are expressed by distinct renal cell populations, linking immune activation (CKLF), broad metabolic activity (ACLY), and proximal tubule solute transport (SLC13A3) to the cellular reorganization that accompanies AKI.

### 3.8. In Vivo Validation of Lactylation-Associated Signatures in Cisplatin-Induced AKI

**Figure 8:**
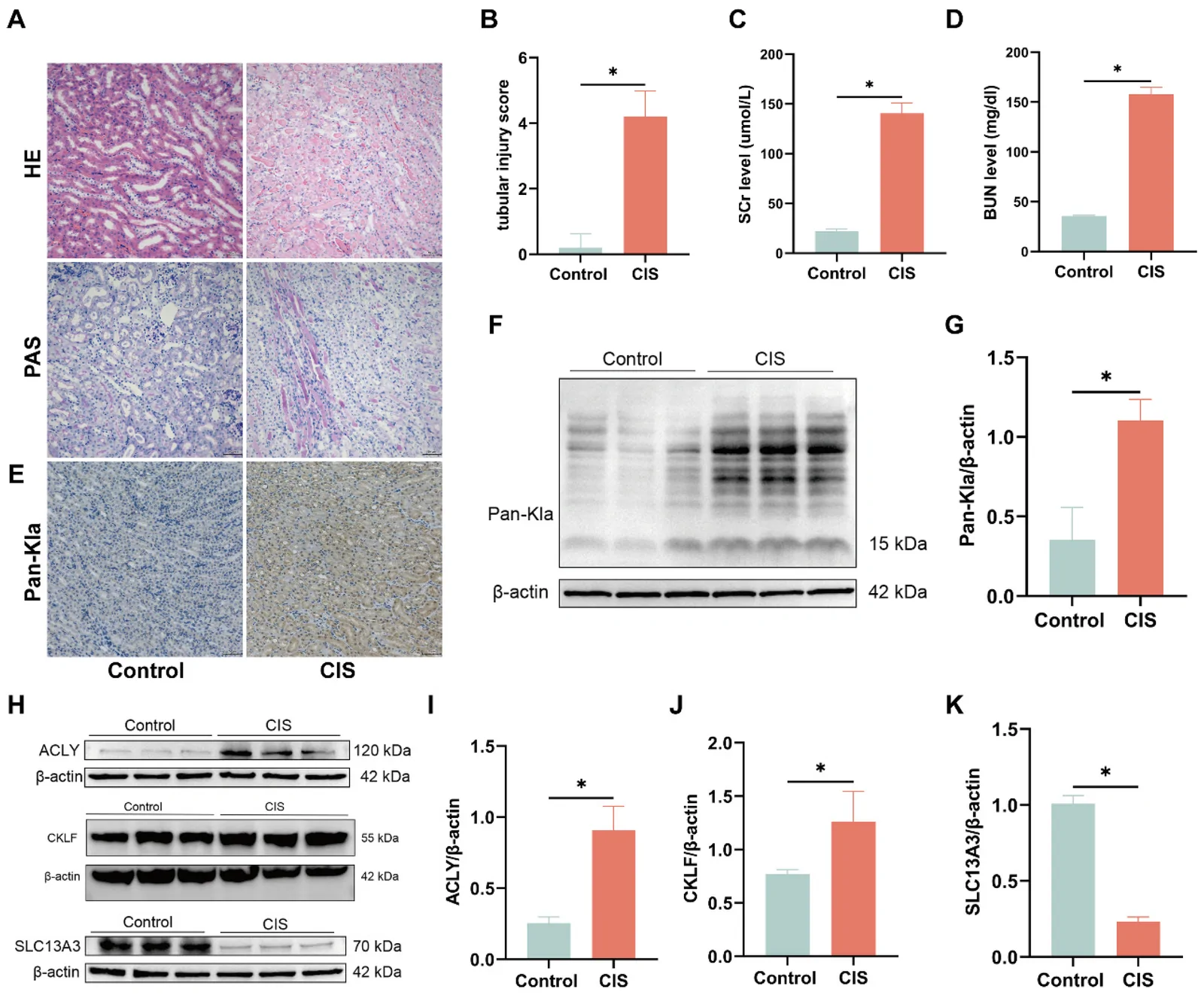
In vivo validation of lactylation-associated signatures in cisplatin-induced AKI. (**A**) H&E and PAS staining of kidney sections. (**B**) Quantification of tubular injury scores. (**C**,**D**) Serum creatinine (Scr) and blood urea nitrogen (BUN) levels in mice. (**E**) Immunohistochemical staining of Pan-Kla in kidney sections. (**F**,**G**) Western blot analysis and quantification of global Pan-Kla levels in kidney tissues. (**H**–**K**) Protein expression levels of ACLY, CKLF, and SLC13A3 in kidney tissues. Data are presented as mean ± SEM; significance is indicated by * *p* < 0.05.

We established a cisplatin-induced acute kidney injury (AKI) model in C57BL/6 mice. H&E and PAS staining showed obvious tubular epithelial injury, tubular dilation, and disruption of renal tubular architecture in the CIS group compared with the control group (Figure 8A). Quantitative analysis further confirmed a significant increase in tubular injury score in the CIS group (Figure 8B). Serum creatinine and blood urea nitrogen levels were also markedly elevated following cisplatin administration, confirming successful AKI model establishment (Figure 8C,D). We then assessed lactylation-related alterations in AKI kidneys. Immunohistochemistry and Western blot analysis showed that global Pan-Kla levels were markedly increased in the CIS group compared with the control group (Figure 8E–G). Moreover, the protein levels of ACLY and CKLF were significantly increased, whereas SLC13A3 was substantially decreased in CIS kidneys (Figure 8H–K). Collectively, these findings indicate increased global lactylation levels and altered expression of lactylation-associated candidate genes in cisplatin-induced AKI.

## 4. Discussion

Acute kidney injury is a clinical syndrome driven by intertwined tubular, vascular, metabolic, and immune mechanisms [28,29,30,31]. Although extensive studies have characterized inflammatory mediators, oxidative stress, mitochondrial dysfunction, and cell death pathways in AKI, the role of lactate-derived post-translational regulation remains insufficiently understood. In the present study, we integrated bulk transcriptomic analysis, weighted gene co-expression network analysis, machine-learning-based feature selection, immune infiltration analysis, single-cell RNA sequencing, and in vivo experimental validation to characterize lactylation-associated molecular alterations in AKI. Four candidate genes (RALYL, CKLF, ACLY, and SLC13A3) were identified through integrative bioinformatics analysis, and CKLF, ACLY, and SLC13A3 were further prioritized as candidate diagnostic signatures. Single-cell analysis revealed distinct cell-type expression patterns of these genes, while animal experiments demonstrated increased global protein lysine lactylation and altered expression of the candidate genes in cisplatin-induced AKI. Collectively, our findings suggest that lactylation-associated transcriptional alterations reflect the immunometabolic remodeling occurring during AKI and provide candidate signatures for further mechanistic investigation.

The initial differential expression and enrichment analyses showed that AKI kidneys exhibited broad activation of immune and inflammatory pathways. The differentially expressed genes were mainly enriched in inflammatory response, humoral immune response, response to bacterium, extracellular matrix organization, complement and coagulation cascades, phagosome formation, cytokine–cytokine receptor interaction, and metabolism-related pathways. These findings are consistent with the current understanding that AKI is not only characterized by acute tubular epithelial injury, but also represents an inflammation- and immune-mediated pathological process. Oxidative and nitrosative signaling may further amplify this inflammatory environment, as excessive nitric oxide production can interact with reactive oxygen species to generate reactive nitrogen species and promote cellular and tissue injury [32]. Injured tubular epithelial cells can release damage-associated molecular patterns, chemokines, cytokines, and extracellular vesicles, thereby promoting the recruitment and activation of innate and adaptive immune cells [33,34]. These immune alterations collectively contribute to the complex inflammatory microenvironment that characterizes AKI. This network-level view is consistent with the emerging concept that inflammatory injury is driven not by isolated mediators but by interconnected signaling circuits in which cytokines, intracellular pathways, and immune-cell interactions collectively shape tissue responses [35]. Consistently, WGCNA identified an AKI-associated red module enriched in defense response, antimicrobial humoral response, regulation of inflammation, cytokine-related signaling, IL-17 signaling, JAK-STAT signaling, PI3K-Akt signaling, and Toll-like receptor signaling. Together, these results indicate that differential expression analysis and co-expression network analysis converged on immune activation, inflammatory signaling, and metabolic remodeling as major molecular features of AKI.

A key novelty of this study is the incorporation of lactylation-associated signatures into the transcriptomic analysis of AKI. Lactylation is a recently recognized post-translational modification that links lactate accumulation to transcriptional and functional regulation. During AKI, renal tubular cells are exposed to hypoxia, mitochondrial dysfunction, ATP depletion, oxidative stress, and inflammatory injury, leading to a metabolic shift from oxidative phosphorylation toward glycolysis and subsequent lactate accumulation [36]. Once regarded merely as an end product of anaerobic metabolism, lactate is now recognized as an active signaling metabolite involved in immune regulation, inflammatory responses, gene transcription, and tissue repair [37]. In this context, protein lactylation may represent one potential molecular link between metabolic stress and renal immune and tubular responses. In a cisplatin-induced AKI mouse model, we observed marked tubular epithelial injury and renal dysfunction accompanied by significantly increased global Pan-Kla levels, as demonstrated by histopathological analysis, renal function tests, immunohistochemistry, and Western blot. However, the present study did not determine the specific lactylated proteins or modification sites involved. Therefore, the increased Pan-Kla levels should be interpreted as evidence of enhanced global protein lactylation rather than direct evidence that CKLF, ACLY, or SLC13A3 are regulated by lactylation.

Among the hub genes identified in this study, CKLF showed a strong association with immune activation. CKLF is a chemokine-like factor involved in leukocyte chemotaxis and inflammatory regulation [38,39,40]. In our bulk transcriptomic analysis, CKLF was upregulated in AKI samples, and this finding was further validated at the protein level in cisplatin-induced AKI kidneys. Single-cell RNA sequencing revealed that CKLF was predominantly expressed in immune cells and tended to increase in AKI kidneys. Immune infiltration analysis further showed that CKLF was positively correlated with activated CD4 memory T cells, M1 macrophages, gamma delta T cells, and monocytes, while negatively correlated with regulatory T cells. These immune populations are closely related to inflammatory amplification, antigen presentation, cytokine production, and tissue damage in AKI. The positive association between CKLF and M1 macrophages is particularly notable, as M1-like macrophages are generally considered pro-inflammatory and can aggravate tubular injury during the early phase of AKI [41]. Consequently, CKLF may represent an immune-associated signature reflecting inflammatory microenvironment changes during AKI.

ACLY was identified as another upregulated hub gene and may represent a metabolic signature in AKI. ACLY encodes ATP citrate lyase, a cytosolic enzyme that converts citrate into acetyl-CoA [42]. Acetyl-CoA is a central metabolite required for lipid synthesis, energy metabolism, and protein acylation. In inflammatory and metabolically stressed cells, ACLY can connect glucose-derived carbon flux to epigenetic and transcriptional regulation [43,44]. In this study, ACLY was upregulated in AKI samples, ranked highly in the random forest model, and was elevated at the protein level in the cisplatin-induced AKI model. Single-cell analysis showed that ACLY was broadly expressed across renal cell types rather than being confined to a single compartment, suggesting that ACLY may reflect a generalized metabolic response to renal injury. In addition, the correlation between ACLY expression and immune cell populations suggests potential interactions between metabolic remodeling and immune alterations during AKI. Considering the close relationship between cellular metabolism and lactylation regulation, increased ACLY expression may reflect metabolic adaptation associated with AKI progression.

In contrast to CKLF and ACLY, SLC13A3 was significantly downregulated in AKI. SLC13A3 encodes the sodium-dependent dicarboxylate cotransporter NaDC3, which is highly expressed in renal proximal tubule cells and mediates the transport of succinate, citrate, and other tricarboxylic acid cycle intermediates [45]. The proximal tubule is one of the most metabolically active nephron segments and is highly susceptible to ischemic and nephrotoxic injury [46]. In our single-cell analysis, SLC13A3 expression was largely restricted to proximal tubular cells and was markedly downregulated in AKI. This expression pattern was corroborated by in vivo validation, which demonstrated diminished SLC13A3 protein levels in cisplatin-induced AKI kidneys. The reduction in SLC13A3 expression likely reflects proximal tubular cell injury, the loss of differentiated transport functions, or metabolic maladaptation. Given that dicarboxylate transport is intimately coupled to mitochondrial metabolism and organic anion handling, the downregulation of SLC13A3 indicates impaired proximal tubular metabolic homeostasis during AKI. The distinct expression patterns of CKLF, ACLY, and SLC13A3 suggest that these candidate signatures capture interconnected different biological aspects of AKI, including immune alterations, metabolic responses, and proximal tubular changes. Specifically, CKLF reflects immune activation and leukocyte-associated inflammation; ACLY reflects broad metabolic reprogramming and acyl-CoA-dependent regulatory capacity; and SLC13A3 mirrors proximal tubular transport function and metabolic integrity. This multidimensional signature may partially explain the improved discriminatory performance of the combined nomogram compared with individual markers in the training cohort. Given its high heterogeneity, AKI is unlikely to be fully characterized by a single molecular axis, as its clinical and pathological manifestations emerge from the complex interplay of multiple biological processes. Therefore, a combined signature integrating inflammatory, metabolic, and tubular components more accurately encapsulates the biological complexity of AKI.

Another notable finding is that consensus clustering based on the four lactylation-associated signatures divided AKI samples into two molecular subtypes. This result indicates that lactylation-associated transcriptional signatures can delineate the underlying disease heterogeneity in AKI. Clinically, AKI is a highly heterogeneous syndrome triggered by diverse etiologies, including ischemia–reperfusion injury, sepsis, nephrotoxicity, major surgery, and systemic inflammation. Even among patients with similar clinical diagnoses, the underlying molecular processes may differ substantially. The identification of lactylation-related subtypes suggests that AKI patients may have distinct metabolic and immune states, which may be associated with differences in disease trajectories and treatment responses. Although the current study lacked sufficient longitudinal clinical data to rigorously evaluate the prognostic significance of these subtypes, this discovery provides a rationale for future studies to explore lactylation-based molecular stratification in AKI.

Several limitations should be acknowledged. First, although multiple public transcriptomic datasets and experimental validation were integrated, the sample sizes of the training and validation cohorts were relatively limited, and differences in experimental platforms and patient characteristics may introduce potential heterogeneity. Second, the machine learning model showed good performance in the training cohort; however, the absence of a prospective independent test cohort and nested cross-validation may increase the risk of model overfitting. Therefore, larger multicenter cohorts are required to further evaluate the robustness of the identified signatures. Third, although increased global protein lactylation was observed in AKI kidneys, we did not directly confirm lactylation modification of CKLF, ACLY, or SLC13A3. Future lactylomic and proteomic studies are needed to elucidate the direct molecular mechanisms. Finally, limited clinical information was available in the public datasets, restricting further evaluation of the clinical applicability of these candidate signatures.

## 5. Conclusions

This study integrated bulk transcriptomic analysis, single-cell RNA sequencing, machine learning approaches, and experimental validation to identify CKLF, ACLY, and SLC13A3 as candidate lactylation-associated signatures in AKI. These signatures reflect distinct aspects of AKI-associated immunometabolic alterations, including immune activation, metabolic adaptation, and proximal tubular alterations. Our findings provide potential molecular candidates for further biomarker evaluation and mechanistic investigation of lactylation-associated molecular alterations in AKI.

## Data Availability

The datasets generated and/or analysed during the current study are available in the Gene Expression Omnibus (GEO) repository, GSE30718 (GEO Accession viewer (nih.gov)), GSE GSE139061 (GEO Accession viewer (nih.gov)), GSE183276 (GEO Accession viewer (nih.gov)).
